# Ink-lithographic fabrication of silver-nanocrystal-based multiaxial strain gauge sensors through the coffee-ring effect for voice recognition applications

**DOI:** 10.1186/s40580-022-00337-3

**Published:** 2022-10-08

**Authors:** Junhyuk Ahn, Hyung Jin Choi, Junsung Bang, Gayeon Son, Soong Ju Oh

**Affiliations:** 1 Department of Materials Science and Engineering, Korea University, Seoul, 02841 Republic of Korea; 2 Department of English Language and Industry, Kwangwoon University, Seoul, 01897 Republic of Korea

**Keywords:** Silver nanocrystals, Coffee-ring effect, Ink-lithography, Multiaxial sensors, Voice recognition, Surface chemistry

## Abstract

Human voice recognition techniques have remarkable potential for clinical applications because information from acoustic signals can reflect human body conditions. This paper reports the fabrication of Ag nanocrystal (NC)-based multiaxial wearable strain gauge sensors by ink-lithography for voice recognition systems. Benefiting from the one-step-device-fabrication strategy of ink-lithography, which can yield Ag NC patterns with specific dimensions and endow physical properties, the Ag NC-based multiaxial strain sensors can be fabricated on an ultrathin substrate (~ 6 μm). Additionally, the coffee-ring effect can be induced onto the Ag NC patterns to realize high sensitivity and angle dependence (gauge factors ${G}_{0^\circ }$ = 11.7 ± 1.2 and ${G}_{90^\circ }$ = 105.5 ± 20.1); moreover, the voice onset time for voice recognition can be detected by the sensors. These features assist in distinguishing between voiced and voiceless plosive contrasts via measurements of contact-based voice onset time differences and can act as a cornerstone for further advancements in wearable sensors as well as voice recognition and analysis.

**Graphical Abstract:**

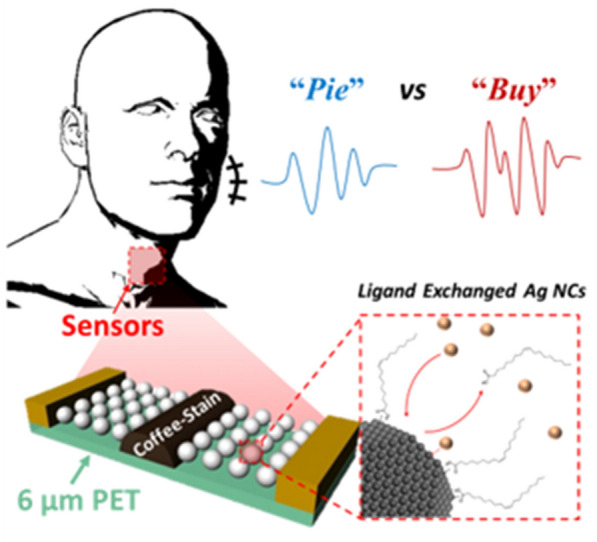

**Supplementary Information:**

The online version contains supplementary material available at 10.1186/s40580-022-00337-3.

## Introduction

The importance of technology for measuring physiological parameters related to human organs, facial muscles, and vocal cords has been increasingly emphasized, particularly in the context of an aging society [1–3]. Continuous investigations of the subtle signals, in particular the human voice, provide clues about various illnesses such as Parkinson’s disease, attention-deficit/hyperactivity disorder, and even COVID-19 [4–7]. In terms of the information that the human voice can carry, certain phonetic features, such as voice onset time (VOT) and fundamental frequency, can be an effective measure for estimating specific body signals. However, common wearable sensors remain insufficient in terms of grasping the acoustic information contained in human speech sounds, although they can be an asset for enabling accurate and fast analyses of human body conditions. In particular, resistive-type strain gauge sensors that detect mechanical deformation by monitoring changes in resistance are known to effectively yield various types of body-related information [8–10]. However, despite their high sensitivity, most sensors require further development to detect human voice and pronunciation because they provide only a monoaxial signal and are constructed on thick substrates, which are unsuitable for attachment to the body.

Colloidal nanocrystals (NCs) are promising materials for high-sensitivity strain gauge sensors owing to their controllable physical properties and solution-based processability [11–13]. However, conventional lithographic techniques, such as photo- and e-beam lithography, cannot be fully utilized for these NCs because of their reactive surfaces, thereby hindering the patterning of NC thin films and realization of sensor arrays, especially on ultrathin substrates [14–16]. This impediment significantly limits multiaxial sensitivity and conformal contact on human skin, such as on vocal cords. However, inkjet printing can be adopted as a potential solution for these problems because it directly and selectively extracts the NC ink for patterning in the absence of harsh conditions [17–19]. Unfortunately, the device performance can be reduced because the NCs in the ink are susceptible to agglomeration or sintering owing to their low colloidal stability [20, 21]. Consequently, a fundamental and comprehensive voice analysis has not been performed using NC-based sensors despite their high sensitivity.

Herein, we report Ag NC-based angle-dependent high-sensitivity strain gauge sensors fabricated through ink-lithography, which selectively prints a surface-modifying ink on Ag NC thin films. Because lithography is conducted under benign conditions, extremely thin substrates can also be employed for subtle motion sensors. Additionally, this method enables the realization of angle-dependent and highly strain-sensitive Ag NC patterns via the control of the printing modes, leading to different surface morphologies. Alternate and continuous (conventional) printing modes were defined, and their corresponding surface morphologies were investigated. The alternate and continuous printing modes result in bulging surfaces via the coffee-ring effect and a uniform surface morphology, respectively [22]. The electromechanical properties of thin films with these surfaces were examined at several angular orientations, and the corresponding gauge factors (*G*) were calculated, with *G* defined as the relative change in resistance per unit strain [23]. The alternately printed patterns on an ultrathin ~ 6 μm-thick polymer substrate showed a *G* value that was approximately 10 times higher (105.5 ± 20.1) than that of the continuous pattern and provided angle dependence. Consequently, the sensors successfully distinguished between voiced and voiceless plosive contrasts by measuring contact VOT differences and successfully detecting the phonetic features for voice recognition.

## Results and discussion

**Scheme 1:**
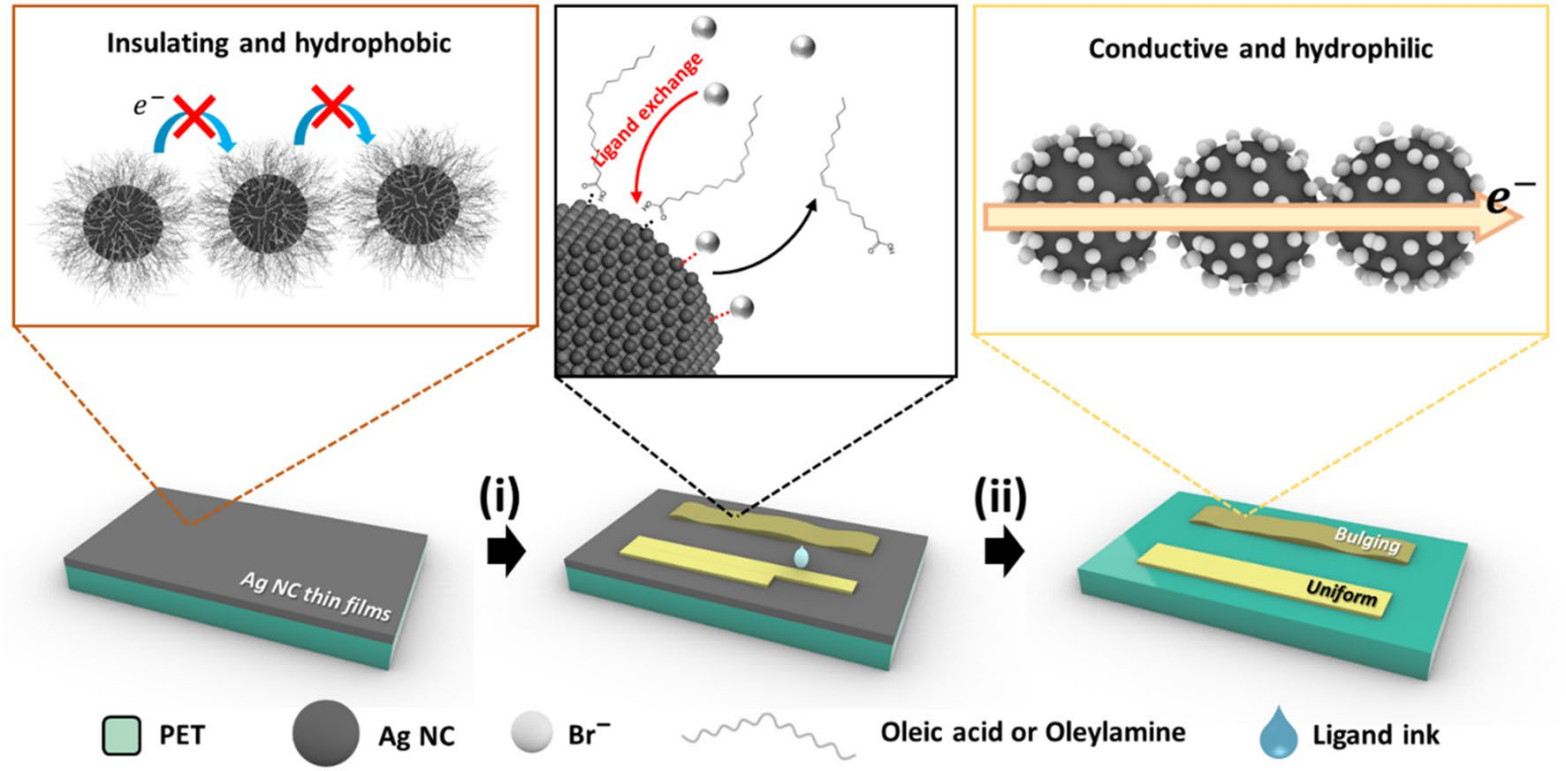
Schematic illustration of the process of ink-lithography and ligand exchange, showing (i) inkjet printing of the ligand ink (NH_4_Br in *iso*-propanol) and (ii) washing with hexane

The as-synthesized Ag NCs were surrounded by the hydrophobic ligands oleic acid and oleylamine, which enabled dispersion of the Ag NCs in hexane, a non-polar solvent, resulting in a colloid. The colloidal Ag NCs were spin-coated as thin films on a polyethylene terephthalate (PET) substrate. Because the long interparticle distance in the Ag NC thin films induces insulating properties, ink-lithography was employed to endow them with electrical characteristics and define the dimensions of the Ag NC patterns, as shown in Scheme 1(i). This method, which was previously reported by our group, is a one-step-device-fabrication strategy that involves achieving selective surface modification via local exposure of the ligand ink on NC thin films [24]. We selected the Br^–^ as a ligand for modifications because the halide ion shows high chemical interaction with Ag NCs and their small size. The exposed area becomes electrically conductive because the ligand ink (NH_4_Br in *iso*-propanol) replaces the oleate ligand with Br^−^ reducing the interparticle distance and facilitating electron transport through the NCs [25]. Additionally, the surface modification makes the Ag NCs hydrophilic, and the untreated hydrophobic Ag NC thin films can be stripped with a nonpolar solvent—hexane [Scheme 1(ii)]. Moreover, different surface morphologies can be realized by controlling the printing mode (Scheme 1, right).

**Fig. 1:**
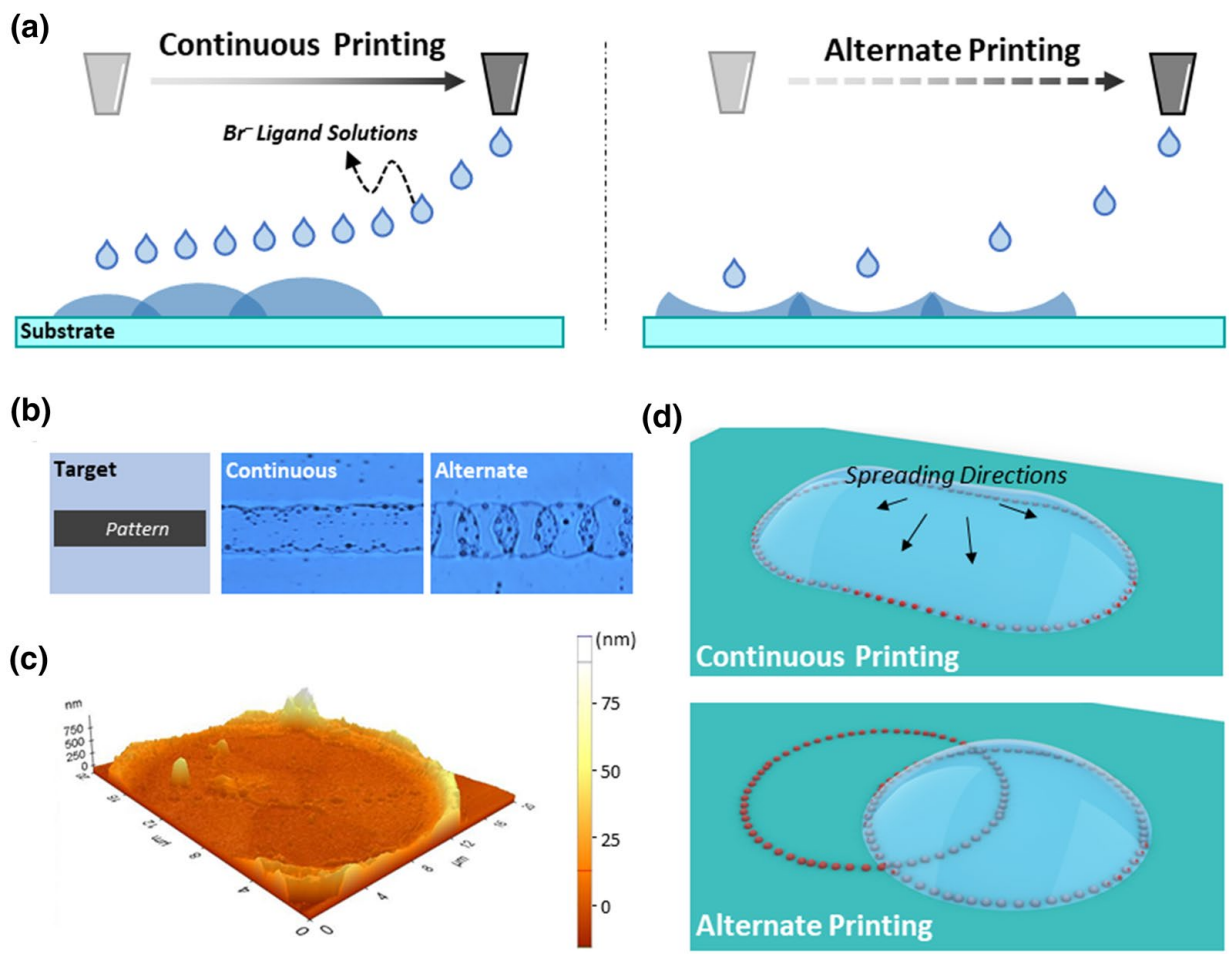
**a** Schematics of different fluid behaviors in the different printing modes (continuous and alternate). **b** Optical images of NH_4_Br-printed glass substrate without NC thin films showing continuously (middle) and alternately printed patterns (right). **c** Atomic force microscopy (AFM) data of an alternately extracted droplet on the glass substrate. **d** Schematic illustration of surface morphology created by the coffee-ring effect (red particles represent oleic acid, oleylamine, or AgBr)

Nanocracks, effective for designing electron movement mechanism, are known to be important for realizing high sensitivity; however, the strategies adopted to date for generating nanocracks are not sufficiently reliable and repeatable because they depend on mechanical deformation or random clustering. Therefore, artificial cracks were generated on the Ag NC thin films in this study by controlling the printing mode. First, the continuous and alternate printing modes were defined, and the surface morphologies induced by them were investigated (Fig. 1a). In the continuous (conventional) printing mode, the ligand ink was extracted onto the substrate at a high frequency (20.0 kHz), resulting in a uniform surface morphology with continuous patterns. In the alternate printing mode, the ligand ink was printed onto the substrate at a low frequency (1.0 kHz), which led to bulging surfaces; the details of each printing mode are described in the Experimental Section and Additional file 1: Figure S2.

To examine the surface morphologies induced by each printing mode, the ligand ink—NH_4_Br dissolved in *iso*-propanol—was extracted as a line pattern onto a glass substrate without the Ag NC thin films (Fig. 1b). The continuous printing mode showed a straight-line pattern (Fig. 1b, middle), whereas the alternate printing mode exhibited periodic circular patterns with overlapping ends (Fig. 1b, right); this behavior was attributed to rapid evaporation (< 0.5 ms) [26]. As the pre-extracted droplets completely evaporated prior to subsequent extraction, the ring-shaped contact line was pinned to the substrate.

The morphologies induced by each printing mode can be explained by the coffee-ring effect [23, 27]. To investigate the stain, the surface morphology induced by a single droplet was investigated by AFM (Fig. 1c and S3). The height of edge corresponding to the droplet stain was estimated to be 45.2 ± 2.0 nm, with that of the center being 8.9 ± 1.2 nm. This morphology, induced by the coffee-ring effect, is typically observed when it shows a low contact angle [28, 29]. Marangoni and capillary flows can be generated in droplets with a low contact angle, such as those of the NH_4_Br ligand ink (< 22; Additional file 1: Figure S4) [30–33]. Different gradients of surface tension or temperature are generated along the center and edge of the droplets. These fluctuations lead to different evaporation rates at the air–liquid interfaces and tend to replenish the interior droplet, leading to fluid flow that subsequently transports the interior particles toward the edge of the droplet. When multiple droplets were continuously extracted onto the substrate, an extended elliptical stain was formed (Fig. 1d, top; red dots), whereas extraction of the droplets with a time interval resulted in a circular stain (Fig. 1d, bottom).

**Fig. 2:**
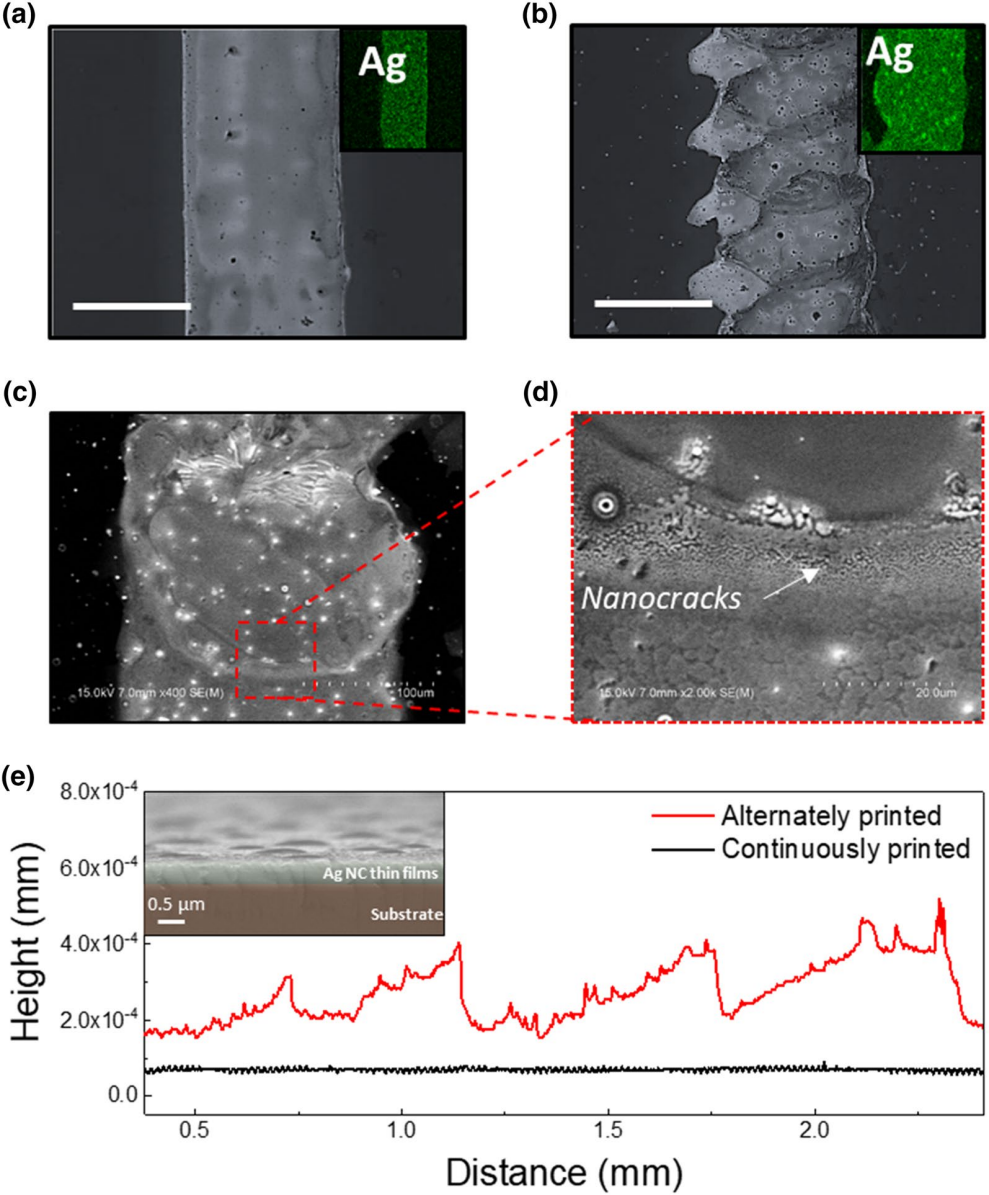
Optical microscopic images of NH_4_Br-treated Ag NC thin films with **a** continuously and **b** alternately printed line patterns (scale bar = 250 μm); insets show energy-dispersive X-ray spectroscopic mapping images for Ag Lα 1. **c** Scanning electron microscopy (SEM) image of an alternately printed line pattern whose magnified version is shown in **d**. **e** Heights of the developed patterns determined using an Alpha-Step profilometer (inset = cross-sectional SEM image of as-deposited Ag NC thin films)

Each printing mode was applied to the Ag NC thin films to fabricate Ag NC patterns, and their surface morphologies were investigated by optical microscopy, energy-dispersive X-ray spectroscopy (EDX), and profilometry (Fig. 2). First, the changes in the characteristics were investigated by performing chemical, optical, and structural analyses (Additional file 1: Figure S1). The results confirmed that the inkjet-printed Ag NC patterns were successfully ligand-exchanged (further details are provided as notes in the Supporting Information). Straight and winding Ag NC patterns were obtained in the continuous and alternate printing modes, respectively, in line with the results shown in Fig. 1 on the different surface morphologies realized for the two printing modes. Straight-line patterns with clean edges and winding patterns were created in the continuous (Fig. 2a) and alternate printing modes (Fig. 2b), respectively. In addition, Ag was detected in each corresponding pattern (insets of Fig. 2a, b). Furthermore, the resolutions of the printing modes on the Ag NC thin films were investigated (Additional file 1: Figure S5a), which indicated that the line widths of the continuous and alternate printing modes were 70.3 and 82.1 µm, respectively. The slightly increased width of the alternately printed Ag NC line pattern was due to the diffusion of the ligand ink into the Ag NC thin films (details of the dependence of resolution on the printing conditions are provided in the Supporting Information).

The Ag NC-based winding-line patterns were investigated via SEM (Fig. 2c). The edge of the circular region showed a brighter gray color than the center, the details of which are highlighted in Fig. 2d. Several white clusters and miniscule black dots, which are insulating regions, were observed. In a typical SEM analysis, white clusters and black dots represent insulating materials and empty space, respectively. This implies that insulating residues such as AgBr and oleate, which were generated via ligand exchange, were transported to the edge of the patterns via the coffee-ring effect. Being nanocracks, these residues were stripped away by a washing process. The surface morphologies are also examined by profilometry and cross-sectional SEM in Fig. 2e. As seen in the inset of Fig. 2e, as-deposited Ag NC thin films show thicknesses of 411 ± 30 nm. As the long ligands of oleic acid and oleylamine were replaced by short inorganic ligand of Br^–^, the thickness of NC thin films was reduced. The thickness of the continuously- and alternately printed Ag NC patterns were obtained to be 85.4 ± 7.2 nm (black line) and 272.1 ± 79.3 nm (red line), respectively. The large thickness and deviation in the height of the alternately printed Ag NC patterns are attributed to the non-uniform surfaces induced by the coffee-ring effect.

**Fig. 3:**
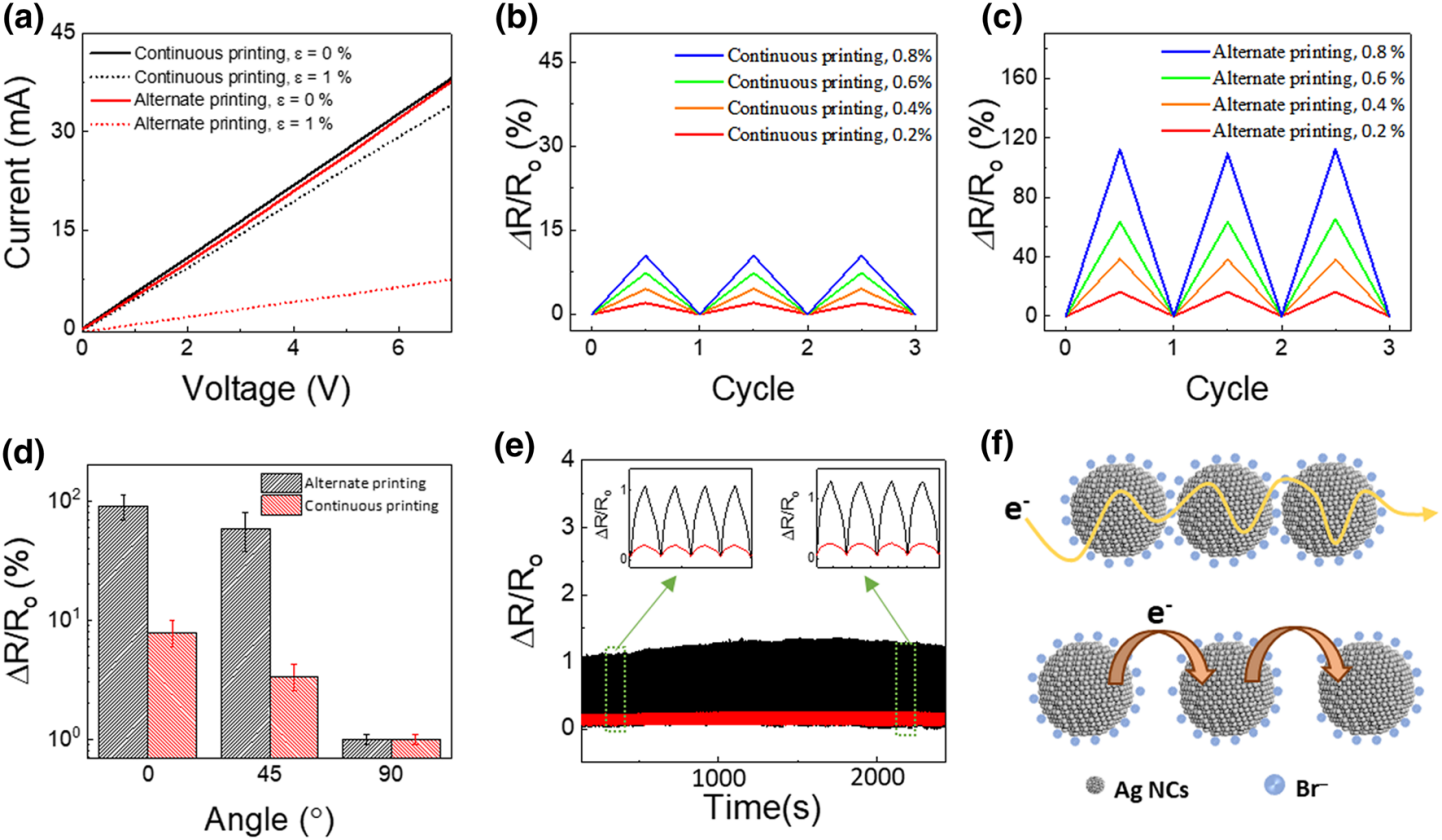
Electrical and electromechanical properties of patterns coated on a 250 μm thick PET substrate. **a** Current–voltage plot of the Ag NC thin films with continuously and alternately printed patterns (solid black and red profiles, respectively) that were strained to 0% and 1% (solid and dot-dash lines, respectively). Changes in resistance of specimens with **b** continuously and **c** alternately printed patterns strained to 0.2% (red), 0.4% (orange), 0.6% (green), and 0.8% (blue). **d** Changes in resistance of specimens strained to 0.8% as a function of angular orientation (0°, 45°, and 90°). **e** Cycle test of specimens strained to 1%; insets show four-cycle properties of coupled (red) and periodic (black) Ag NC patterns. **f** Schematic illustration of electron movement mechanism in scenarios with different interparticle distances

*I*–*V* curves were constructed, and changes in the resistance of the patterns under strain were examined to investigate the dependence of the electrical and electromechanical properties of the thin films on their surface morphology (Fig. 3). The electrical conductivities of both patterns were examined at strains (ε) of 0 and 1% (Fig. 3a), which were found to be linearly proportional to the applied voltage. The average resistance of the continuously and alternately printed patterns changed from 11.1 ± 0.1 to 12.0 ± 0.3 kΩ and from 45.2 ± 1.4 to 106.0 ± 55.4 kΩ, respectively. Before and after bending, the conductivity of the continuously printed Ag NC patterns was higher than that of the alternately printed counterparts. The low conductivity of the alternately printed Ag NC patterns is attributed to the nanocracks shown in Fig. 2d.

Subsequently, electromechanical properties of the thin films were measured with respect to their printing mode (Fig. 3b, c). By conducting a three-cycle test, the sustainability and changes in resistance were investigated at strains of 0.2, 0.4, 0.6, and 0.8%. The resistance changes (ΔR) is defined as the difference between the resistance of a strained specimen (R_*i*_) and its initial resistance (R_o_). The continuously and alternately printed Ag NC patterns showed changes in resistance (ΔR/R_*i*_) of 2.0, 4.5, 7.4, and 10.5% and 16.4, 38.8, 63.6, and 109.6% at the aforementioned strains, respectively. The corresponding gauge factor was calculated as

1 $${\text{G }} = \, \left( {\Delta {\text{R}}/{\text{R}}_{{\text{o}}} } \right)/\varepsilon$$

The average gauge factors of the continuously and alternately printed Ag NC patterns were estimated to be 11.7 ± 1.2 and 87.2 ± 10.1, respectively.

The changes in resistance of samples strained to 1.0% at different angular orientations (0°, 45°, and 90°) were subsequently determined (Fig. 3d, Additional file 1: Figure S6and S7). The change in resistance of the continuously printed Ag NC patterns slightly decreased to 7.91 ± 2.0, 3.4 ± 0.8, and 0.9 ± 0.1 for the angular orientations of 0°, 45°, and 90°, respectively, whereas the alternately printed counterparts showed corresponding values of 91.1 ± 12.8, 59.0 ± 2.7, and 1.1 ± 0.1. The changes in the resistance of both patterns were reduced upon rotation to 90°; however, the alternately printed Ag NC patterns showed a more considerable angle-dependent strain sensitivity than that of the continuously printed equivalents; this sensitivity can be attributed to the strained nature of the electrically conductive Ag NC patterns. While the electromechanical properties of the continuously printed Ag NC patterns were mainly influenced by the bending-induced increase in the interparticle distance between the Ag NCs, the alternately printed counterparts were influenced by angle-dependent changes in the width of the stains and the lengthened interparticle distance. The different angle-independent strain sensitivities of the two Ag NC patterns are elaborated further in the Supporting Information and Additional file 1: Figure S7.

Device sustainability was subsequently examined by conducting a 1000-cycle test using samples strained to 1.0% (Fig. 3e). Both the continuously and alternately printed Ag NC patterns showed consistent changes in resistance up to 1000 cycles. Both patterns maintained their initial electromechanical properties until the end of the strain tests (Fig. 3e, insets). The gauge factor and stability with the cycle test at high bending strain is also discussed in the Supporting Information (Additional file 1: Figure S8). The hysteresis of the strain sensor is examined by monitoring the strain (Additional file 1: Figure S9). The data shows the symmetric curves of resistance changes, implying that both alternately- and continuously printed Ag NC patterns show almost no hysteresis. Overall, the stain created by the alternate printing mode was presumed to considerably increase the interparticle distances via the condensing nanocracks, leading to a high strain sensitivity. This is because the stain and nanocracks induced by the lengthened interparticle distances were micro- and nanoscale in size, respectively. This assumption was reasonable because the electrical conductivity of the NC thin films was closely related to the interparticle distance (*l*) (Fig. 3f). The equation connecting conductivity and interparticle distance can be expressed as

2 $$\sigma \, = \, \sigma_{{\text{o}}} \cdot{\text{e}}^{{ - \beta \cdot{\text{l}}}} ,$$

where σ_o_ is the pre-exponential constant, and β is the tunneling decay constant [34, 35]. When the interparticle distance of NCs is lengthened by long surface ligands or nanocracks, the transport mechanism of tunneling and/or hopping becomes dominant, leading to a low conductivity [36]. Inserting Eq. 2 into Eq. 1 results in an expression that confirms the relationship between interparticle distance and strain sensitivity. Therefore, because the electromechanical properties of the alternately printed Ag NC patterns changed with different stain lengths (Fig. 3d and Additional file 1: Figure S7), the stains were confirmed to be advantageous for achieving high sensitivity.

**Fig. 4:**
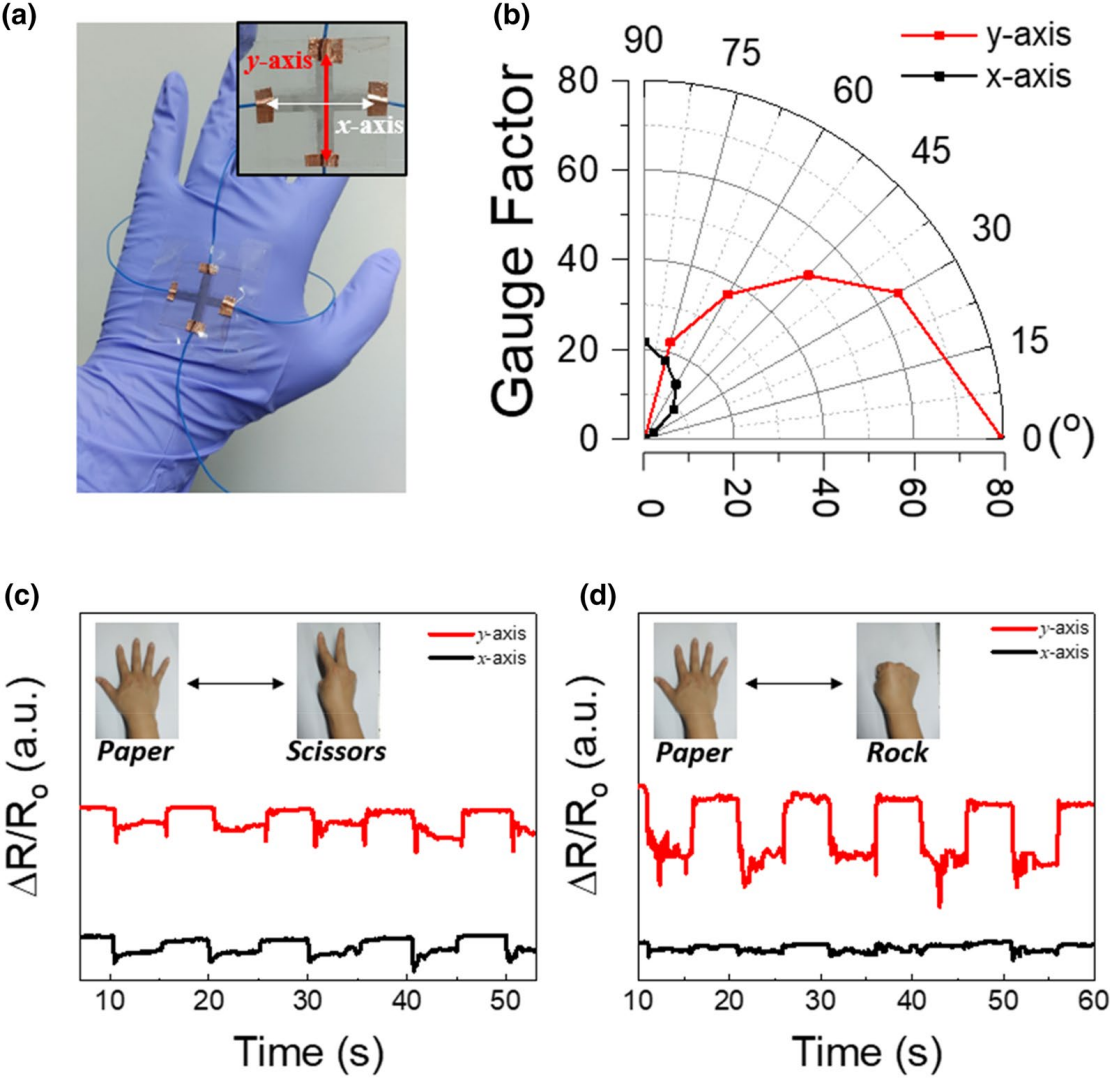
**a** Optical image of multiaxial strain gauge sensors on the hand (inset illustrates the *x*- and *y*-axes of the attached strain gauge sensors). **b** Effective gauge factors of the *x*- and *y*-axis-oriented sensors in a cross-shaped device as a function of angle from the *x*-axis. Variations in device resistance with changes in shape based on the **c** *scissors* and **d** *rock* configurations

Multiaxial strain gauge sensors for human motion detection were subsequently fabricated in the different printing modes (Fig. 4), and the *x*- and *y*-axis-dependent detection was demonstrated. The *x*- and *y*-axis sensors were fabricated using the continuously and alternately printed Ag NC patterns, respectively (Fig. 4a). The constructed sensing device was attached to the back of the hand, and electrical signals were obtained by wiring the electrode at the end of each sensor. The manufacturing details of the multiaxial strain sensors are provided in the Experimental Section.

The effective gauge factor (${G}_{i}^{*}$), which can reflect angle-dependent mechanical deformation in the 0°–90° range, was subsequently investigated (Fig. 4b). The average ${G}_{x}^{*}$ showed increments of 2.5, 9.2, 14.0, 18.0, and 21.5 in an anticlockwise manner. In contrast, the average ${G}_{y}^{*}$ showed decreases of 79.7, 65.2, 51.6, 37.1, and 22.3 in a clockwise manner. These results indicate that the multiaxial strain sensors could predict angle-dependent mechanical deformation by estimating the electrical signals corresponding to each axis.

The prediction of hand motion using the multiaxial strain gauge sensors was subsequently demonstrated (Fig. 4c, d). Essentially, the sensor performance was examined by monitoring the electrical signals corresponding to the hand motions of *rock*–*paper*–*scissors*. The hand shapes corresponding to *paper*, *scissors*, and *rock* represent flat, spherical, and monoaxial curved surfaces, respectively. The changes from the *paper* to *scissors* configurations entailed the simultaneous curvature of the *x*- and *y*-axes, which led to an increase in the resistance in both axes (Fig. 4c). In the *paper*-to-*rock* transformation, the *y*-axis-oriented Ag NC patterns were bent to a greater degree than the *x*-axis counterparts, which led to greater changes in resistance in the *y*-axis-oriented Ag NC patterns (Fig. 4d). Therefore, the hand motions of *rock*–*paper*–*scissors* were successfully classified by monitoring the electrical signals of the two axes.

**Fig. 5:**
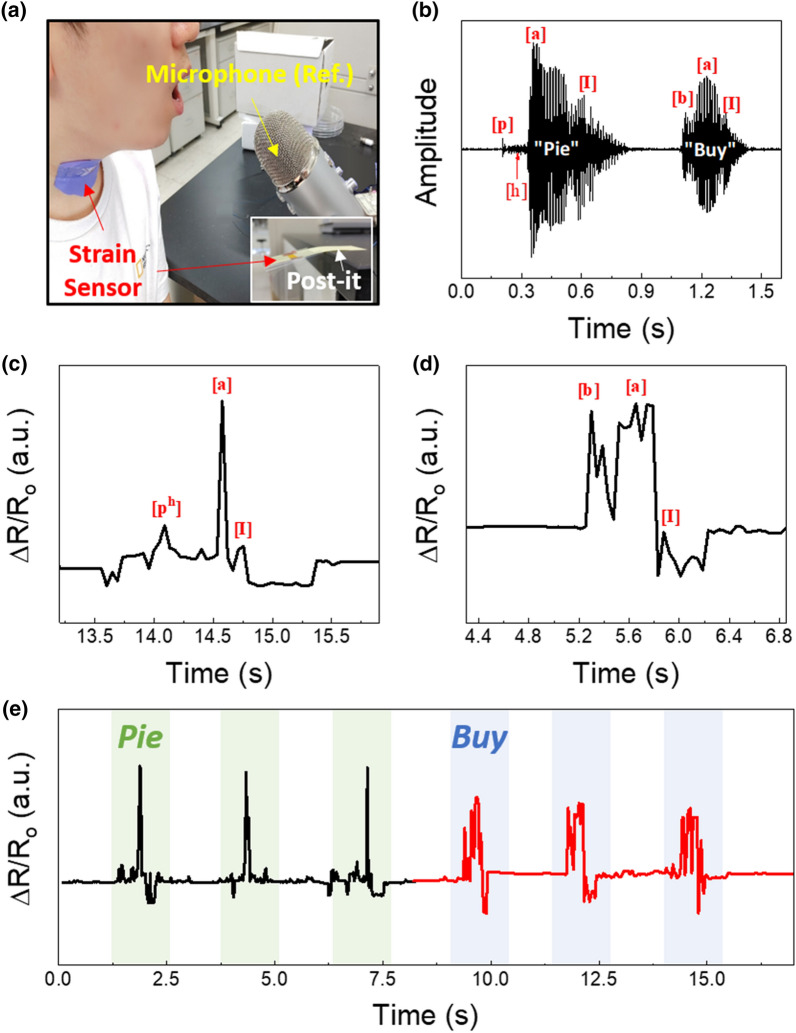
**a** Photograph of the alternately printed Ag NC thin films attached to the neck of a speaker; inset shows a photograph of the strain sensors on a Post-it note. **b** Phonation waveforms of *pie* and *buy* recorded using a reference microphone. Changes in resistance of alternately printed Ag NC thin films attached to the neck for phonating **c** *pie* and **d** *buy*. **e** Repetition test for productions of *pie* and *buy*

Voice recognition techniques directly pertain to acoustic feature detection. For voicing pairs in English, the length of the delay in vocal cords vibration, which is known as VOT, is considered a key cue for distinguishing between voiced and voiceless plosive production. The voiced plosive /d/ and its voiceless counterpart /p/ require different articulatory gestures for pronunciation, and the difference in VOT between them shows phonetic differences. As shown in Fig. 5, different types of phonation signals were tested using the alternately printed Ag NC thin films. To assemble the voice recognition system, the colloidal Ag NCs were coated on an ultrathin 6 μm-thick PET substrate (inset of Fig. 5a). The alternately printed Ag NC thin films were attached to the Adam’s apple of a speaker, which has vocal cords on the inside, to measure the changes in resistance; moreover, the device performance was compared with that of a reference microphone (Fig. 5a).

The changes in amplitude due to productions of *pie* and *buy* were monitored using the microphone (Fig. 5b). The production of *pie* consists of a voiceless aspirated consonant/p/and the vowel/aɪ/, whereas that of *buy* consists of the voiced counterpart/b/and the vowel/aɪ/ [37, 38]. In the case of the production of *pie*, aspiration with a noise burst occurs after the word-initial/p/, followed by small semi-random variations. During the phonation of the vowel/aɪ/, the intensity abruptly increases and gradually drops. Moreover, *buy* is pronounced with a high initial amplitude after the/b/without strong aspiration. The major difference between the two words is the existence of small ranges of variation induced by aspiration.

The changes in resistance of the alternately printed Ag NC thin films induced by the productions of *pie* and *buy* were measured (Fig. 5c, d). The/p/sound enhanced the changes in resistance, and immediately, a small variation occurred with aspiration (Fig. 5c). Moreover, the electrical signal strongly fluctuated with the release of the vowel/aɪ/. In the case of *buy*, changes in resistance were observed at the start of/b/, which decreased after the production of the vowel (Fig. 5d). Therefore, the alternately printed Ag NC thin films on the thin PET substrate were capable of classifying unvoiced and voiced sounds. Moreover, analysis of the performance sustainability (Fig. 5e) indicated that the electrical signals corresponding to repetitive pronunciations of *pie* and *buy* were well-maintained. Consequently, the strategy reported herein is anticipated to contribute significantly to the development of voice recognition systems.

## Conclusion

We report a strategy for the fabrication of Ag-NC-based multiaxial strain gauge sensors via surface morphological engineering of Ag NC thin films. The coffee-ring effect was intentionally induced using an ink-lithography-enabled mode to realize *x*- and *y*-axis-dependent gauge factors. Consequently, multiaxial strain gauge sensors capable of operating in a wide range of angular orientations (0°–90°) were realized. Moreover, voiced and unvoiced sounds were classified using electrical signals, along with the hand motions of *rock*–*paper*–*scissors*. We believe that this strategy will considerably facilitate the utilization of the coffee-ring effect and advance the development of wearable healthcare systems.

## Experimental section

*Chemicals*: AgNO_3_ (ACS, 99.9 + %, metal basis), oleic acid (OA; tech., 90%), and (3-mercaptopropyl)trimethoxysilane (MPTS, 95%) were purchased from Alfa Aesar. Oleylamine (OAm; 70%) was purchased from Sigma-Aldrich. Ammonium bromide (NH_4_Br; ACS reagent,  > 99.0%) was purchased from Honeywell Fluka. Toluene (99.5%), ethanol (95.0%), and isopropanol (IPA; 99.5%) were purchased from Samchun. PET films with thicknesses of 50–250 μm (SKC) were used as the flexible substrate.

### Synthesis of 3–5-nm-sized Ag NCs

AgNO_3_ (1.7 g) was placed in a 100 mL three-necked flask and blended with OA (45 mL) and OAm (5 mL). The container was subsequently degassed for more than 90 min at 70 °C and then filled with nitrogen gas. The flask was heated to 180 °C at 1 °C/min thereafter. When the temperature reached 180 °C, the heating mantle was removed and the solution was cooled to 25 °C. The obtained Ag NC dispersion was divided between two 50 mL centrifuge tubes and mixed with toluene (20 mL). The solution was centrifuged at 8000 rpm for 1 min, after which EtOH was added to the resulting solution and centrifuged further at 5000 rpm for 5 min. After the centrifugation, Ag NCs were collected and dispersed in toluene. Purification was conducted twice, and the Ag NCs were finally re-dispersed in hexane.

### Fabrication of Ag NC thin films

The PET substrates were washed with acetone, IPA, and deionized water, and subsequently UV-treated with UV/ozone. The UV-treated substrates were submerged in MPTS for at least 6 h. The colloidal NCs were spin-coated onto the MPTS-treated substrates with dimensions of 2.54 × 2.54 cm^2^.

### Printing settings

A specific waveform was designed to extract the IPA-based ligand solution (Additional file 1: Figure S2). For the continuous and alternate printing modes, drop spacings of 10 and 30 μm and maximum jetting frequencies of 20.0 and 1.0 kHz, respectively, were adopted.

### Fabrication of strain gauge sensors

Ligand ink (30 mM NH_4_Br in IPA) was inkjet printed on Ag NC thin films. After the ligand solution was printed following the input digital patterns, the untreated region in the surrounding oleate ligands was fully stripped with the hydrophobic solvent hexane. An electrode was connected to the end of each pattern using silver paste and copper tape.

### Characterization

Chemical analysis was conducted via FTIR spectroscopy (LabRam ARAMIS IR2, Horiba Jobin Yvon), and the optical properties (photoluminescence and absorbance) were analyzed using a UV/VIS/NIR spectrophotometer (JASCO, V-770). Structural analysis was conducted using an X-ray diffractometer (D/MAX-2500 V, Rigaku), stylus surface profiler (Kosaka Lab., Alpha-Step ET200), and atomic force microscope (PSIA, XE150). The electrical and electromechanical properties were analyzed using a probe station (MS TECH, model M5VC; Keithley, 4200 and 2400). The surface was analyzed by SEM (High-Tech America, Inc., Hitachi S-4300) and SEM–EDS (TESCAN, LYRA3 XMH). The acoustic sounds were measured using a commercial microphone (Blue Microphones, Yeti STUDIO Silver).

## Supplementary Information

**Additional file 1: Figure S1.** HRTEM images of **a** as-synthesized (left) and NH_4_Br-treated (right) Ag NCs. **b** UV–vis absorbance spectra, **c** FT-IR absorption profiles, and **d** XRD patterns of the as-synthesized (black) and NH_4_Br-treated Ag NCs with continuously (blue) and alternately printed patterns (red).** Figure S2.** Profile of waveform employed for inkjet printing the ligand ink. **Figure S3.** Plot of the AFM data corresponding to the Fig. 1c results. **Figure S4.** Optical image for investigating contact angle of the ligand ink on the Ag NC thin films.** Figure S5.** Optical images of ligand-ink-treated Ag NC line patterns. **a** Continuously (left) and alternately printed (right) Ag NC line patterns. **b** Changes in line width of the Ag NC line patterns with different micro-spacings of the jetting droplets (scale bar = 50 μm).** Figure S6.** **a** Front- and **b** top view optical images of the multiaxial strain gauge sensors attached to the 0.6%-strain-curved structure. **Figure S7.** **a** Schematic of films with alternately printed Ag NC patterns subjected to bending at different rotations. **b** Detailed schematic of alternately printed Ag NC patterns. Detailed top-view schematics of changes in the **c** alternately and **d** continuously printed Ag NC patterns with bending. **Figure S8. a **Gauge factor of alternately- (black dots) and continuously printed Ag NC patterns upon high bending strain. **b** Cycle test of alternately printed Ag NC patterns (upper = 1% strain; lower = 5% strain). **Figure S9**. Hysteresis plot of both Ag NC patterns with 1.0 % strain applied (filled circles or triangles) and released (vacant circles or triangles).
