# Supplementary material for: Ink-lithographic fabrication of silver-nanocrystal-based multiaxial strain gauge sensors through the coffee-ring effect for voice recognition applications
Source: Nano Converg. 2022 Oct 8;9:46. doi: 10.1186/s40580-022-00337-3 (PMC9547562; doi:10.1186/s40580-022-00337-3)
Supplement: Supplementary file 1 — Additional file 1: Figure S1. HRTEM images of a as-synthesized (left) and NH4Br-treated (right) Ag NCs. b UV–vis absorbance spectra, c FT-IR absorption profiles, and d XRD patterns of the as-synthesized (black) and NH4Br-treated Ag NCs with continuously (blue) and alternately printed patterns (red). Figure S2. Profile of waveform employed for inkjet printing the ligand ink. Figure S3. Plot of the AFM data corresponding to the Fig. 1c results. Figure S4. Optical image for investigating contact angle of the ligand ink on the Ag NC thin films. Figure S5. Optical images of ligand-ink-treated Ag NC line patterns. a Continuously (left) and alternately printed (right) Ag NC line patterns. b Changes in line width of the Ag NC line patterns with different micro-spacings of the jetting droplets (scale bar = 50 μm). Figure S6. a Front- and b top view optical images of the multiaxial strain gauge sensors attached to the 0.6%-strain-curved structure. Figure S7. a Schematic of films with alternately printed Ag NC patterns subjected to bending at different rotations. b Detailed schematic of alternately printed Ag NC patterns. Detailed top-view schematics of changes in the c alternately and d continuously printed Ag NC patterns with bending. Figure S8. a Gauge factor of alternately- (black dots) and continuously printed Ag NC patterns upon high bending strain. b Cycle test of alternately printed Ag NC patterns (upper = 1% strain; lower = 5% strain). Figure S9. Hysteresis plot of both Ag NC patterns with 1.0 % strain applied (filled circles or triangles) and released (vacant circles or triangles). [file 40580_2022_337_MOESM1_ESM.docx]

Supporting Information

Ink-lithographic fabrication of silver-nanocrystal-based multiaxial strain gauge sensors through the coffee-ring effect for voice recognition applications

Junhyuk Ahn, Hyung Jin Choi, Junsung Bang, Gayeon Son* and Soong Ju Oh*

**Discussion**

**Investigation of Ag NCs and Ag NC Thin Films Obtained via Surface Modification**

The replacement of oleate ligands was confirmed by high-resolution transmission electron microscopy (HR-TEM), UV–vis spectrophotometry, Fourier-transform infrared (FT-IR) absorption analysis, and X-ray diffraction (XRD). HRTEM analysis of the Ag NC size (**Fig. S1a**) indicated that the size of as-synthesized Ag NCs (4.0 ± 0.2 nm) increased to 19.3 ± 3.8 nm upon ligand exchange with Br^−^. Changes in the interparticle distances induced by the ligand exchange were examined by collecting UV–vis spectra (**Fig. S1b**). An absorption peak was observed at 430 nm in the spectrum of the as-synthesized Ag NC thin films, which corresponded to the localized surface plasmon resonance peak of Ag NCs. Upon ligand exchange with Br^−^, the peak center red-shifted and broadened, indicating that the interparticle distance was shortened by the ligand exchange with small ligands or agglomeration and sintering via Ostwald ripening [1].

Chemical and structural analyses were also conducted to confirm the replacement of the initial oleate ligands with Br^−^ (**Fig. S1c,d**). The as-synthesized Ag NC thin films showed high-intensity peaks in the range of 2800–3200 cm^−1^, which corresponded to the CH– stretches of the oleate ligands (**Fig. S1c**). In contrast, the NH_4_Br-treated Ag NC thin films with the two constructed patterns did not exhibit any noteworthy peaks in the aforementioned range. This suggested that almost all the initial ligands were removed after the NH_4_Br treatment. Structural analysis was also performed to investigate the connections between the Br^−^ ligands and Ag NC surfaces (**Fig. S1d**). The as-synthesized Ag NCs exhibited a diffraction peak at 39.4°, which represents the Ag NCs with the (111) facet. The XRD peaks of the NH_4_Br-treated Ag NCs appeared at 26.9°, 31.1°, 44.5°, and 55.3°, which corresponded to AgBr. These results indicated that the oleate ligands were completely substituted with Br^−^ ligands after the NH_4_Br treatment.

**Investigating the Resolution of Ag NC Patterns**

The micro-spacing of jetting droplets was tuned to improve the patterning resolution, as it is a key factor for defining the printing mode (**Fig. S5b**). The micro-spacing was adjusted from 10 to 50 μm at a constant extraction frequency of 20.0 kHz. The width of the Ag NC line pattern decreased to 35 μm at a microspacing of 40 μm. When the microspacing was set above 50 μm, each Ag NC pattern was transformed into a separated circular design, which enabled each conductive Ag NC pattern to be electrically uncoupled. Indeed, the resolution of 35 μm is similar to our previously reported high resolution of the ink lithography technique, in which the minimum feature size of the line width and line spacing were 35.1 ± 1.2 and 72.1 ± 11.0 μm, respectively [2].

**Investigating the Angle-Dependent Electrical and Electromechanical Properties of the Ag NC Patterns**

The angle-dependent patterns and changes in electron paths of the alternately printed Ag NC patterns were examined to determine the key factor for ensuring their angle dependence (**Fig. S7**). **Fig. S7a** shows a schematic of the bending tests conducted on the alternately printed Ag NC patterns at various rotations (0°, 0° < θ < 90°, and 90°). The stains consisting of residue and voids were simply expressed as black bars, which assisted in hampering the electron transport to the opposite electrodes (**Fig. S7b**). The electrons were assumed to traverse via the shortest route between the conductive Ag NC patterns, and the bending of the substrate was assumed to increase the width of the stain (**Fig. S7c**).

When the substrate coated with the Ag NC thin films was bent at a rotation of 0°, the width (*l*) of the stain increased in the same direction as the electron transport, enhancing the resistance [Eq. 2]. When the substrate was rotated by θ and bent, the vertical width of the stain increased (Δ*l* × cos θ). Because θ ranged between 0° and 90°, cos θ was less than 1.0, and the rotation of the substrate led to a lower gauge factor. Bending at a 90° rotation increased the width of the stain in the horizontal direction, and barely extended it in the direction of electron transport. Therefore, the angle-dependent sensitivity of the alternately printed Ag NC patterns, as illustrated in **Fig. 3d**, can be attributed to the rotation-induced changes in the strain width.

Additionally, the rotation-influenced changes in the electron path of the continuously printed Ag NC patterns were examined (**Fig. S7d**). The bending substrate was assumed to generate nanocracks by increasing the interparticle distances of the Ag NCs. At 0°, the mechanical deformation extended across the width of the nanocracks, thereby increasing the electron transport distance. In contrast to the alternately printed Ag NC patterns, the rotation of the continuously printed Ag NC patterns by θ° rarely induced changes in the electron transport distance, because the shortest distance of electron transport was orthogonal to the nanocracks. Moreover, the 90° rotation did not induce any changes in resistance because the electron transport direction was parallel to that of the nanocracks. Therefore, the stain induced by the coffee-ring effect was confirmed to be advantageous for endowing the films with angle-dependent electrical and electromechanical properties.

**Investigation of electromechanical analysis upon high bending strain**

Both continuously- (red dots) the alternately (black dots) printed Ag NC patterns show changes in gauge factor upon a high bending strain. Each sensor shows stable behavior with gauge factor around 22 and 80 when 1, 2, and 3 % strain is applied, respectively (**Fig. S8a**). However, when 5 % strain is applied, the gauge factor and standard deviation are dramatically increased, representing low reliability. In addition, we conducted the cycle tests to monitor the changes in resistance of the alternately printed Ag NC patterns upon 1% and 5% strain (**Fig. S8b**). When the 1% strain was applied, the changes in resistance show almost constant (**Fig. S8b**, upper). In contrast, the changes in resistance gradually increased upon 5% bending strain (**Fig. S8b**, lower). We attributed this to the permanent damage by enlarged cracks upon high bending strain (>5%).

**
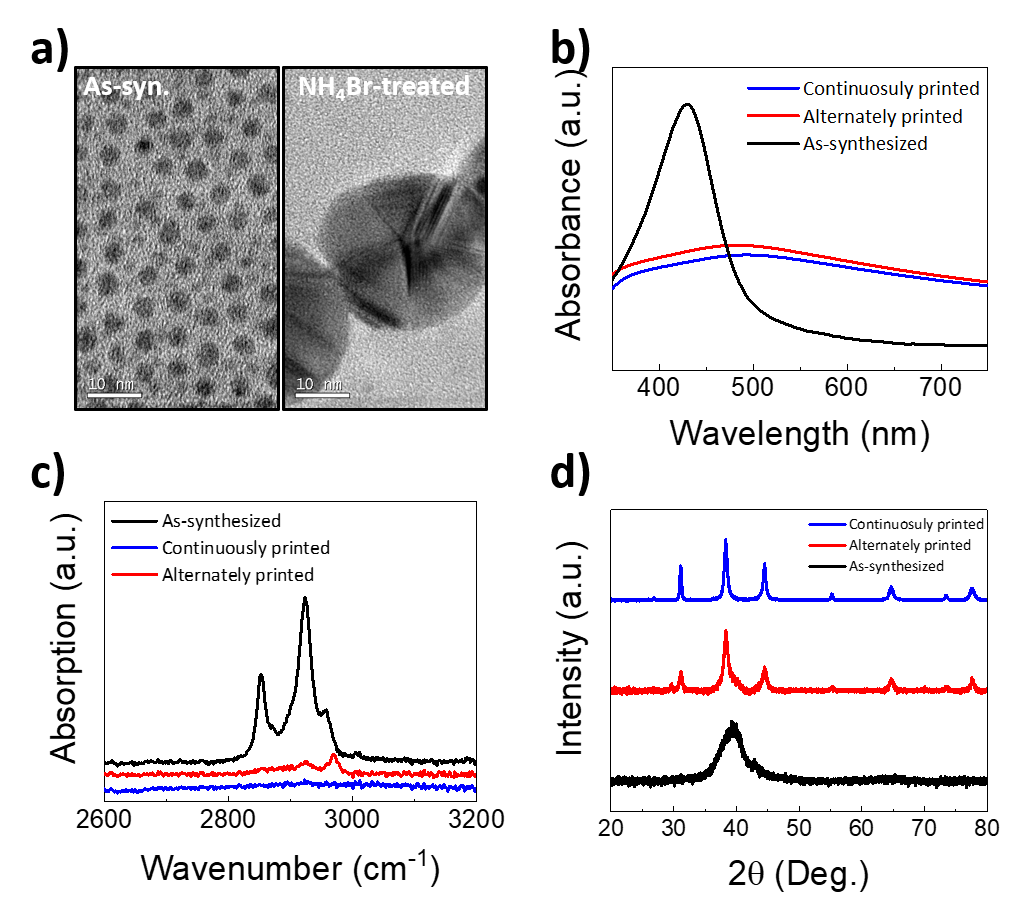
Fig. S1** HRTEM images of **a** as-synthesized (left) and NH_4_Br-treated (right) Ag NCs. **b** UV–vis absorbance spectra, **c** FT-IR absorption profiles, and **d** XRD patterns of the as-synthesized (black) and NH_4_Br-treated Ag NCs with continuously (blue) and alternately printed patterns (red).

**
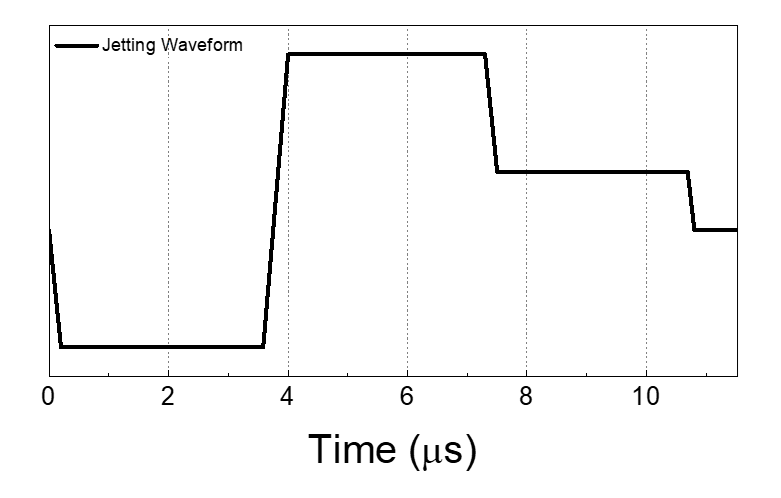
Fig. S2** Profile of waveform employed for inkjet printing the ligand ink.

**
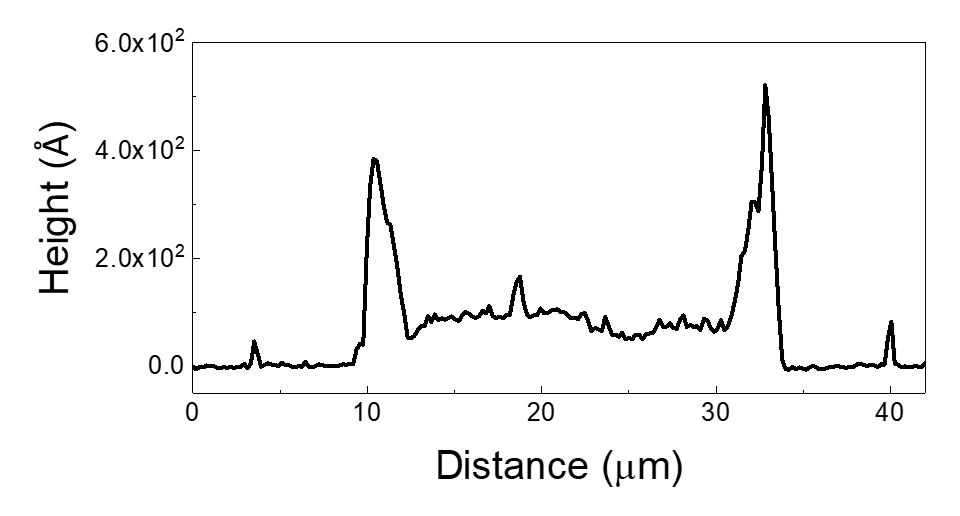
Fig. S3** Plot of the AFM data corresponding to the **Fig. 1c** results.

**
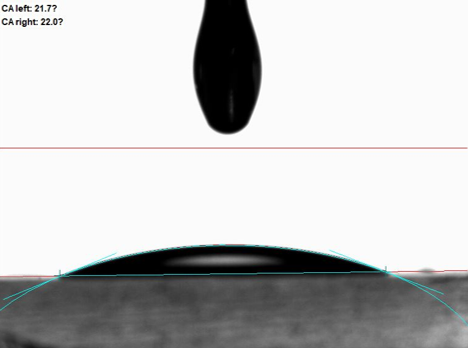
Fig. S4** Optical image for investigating contact angle of the ligand ink on the Ag NC thin films.

**
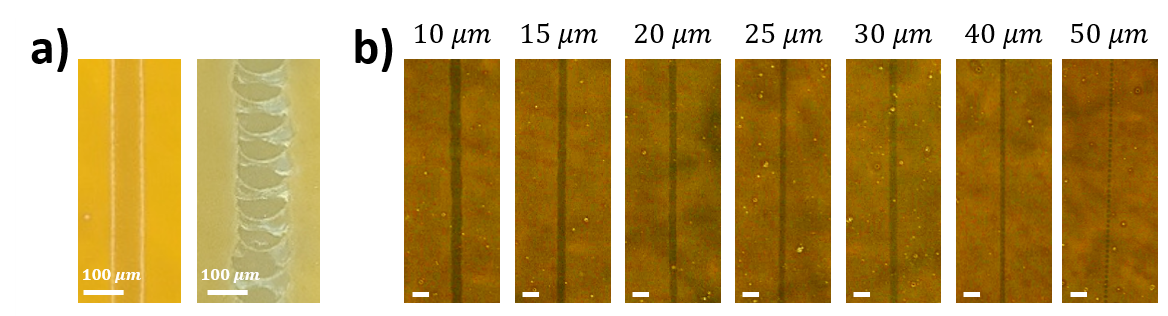
Fig. S5** Optical images of ligand-ink-treated Ag NC line patterns. **a** Continuously (left) and alternately printed (right) Ag NC line patterns. **b** Changes in line width of the Ag NC line patterns with different micro-spacings of the jetting droplets (scale bar = 50 μm).


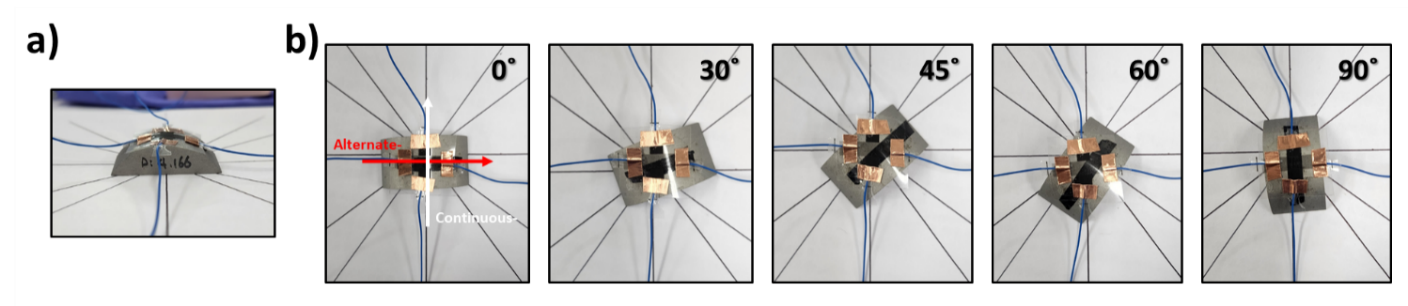
**Fig. S6.** **a** Front- and **b** top view optical images of the multiaxial strain gauge sensors attached to the 0.6%-strain-curved structure

**
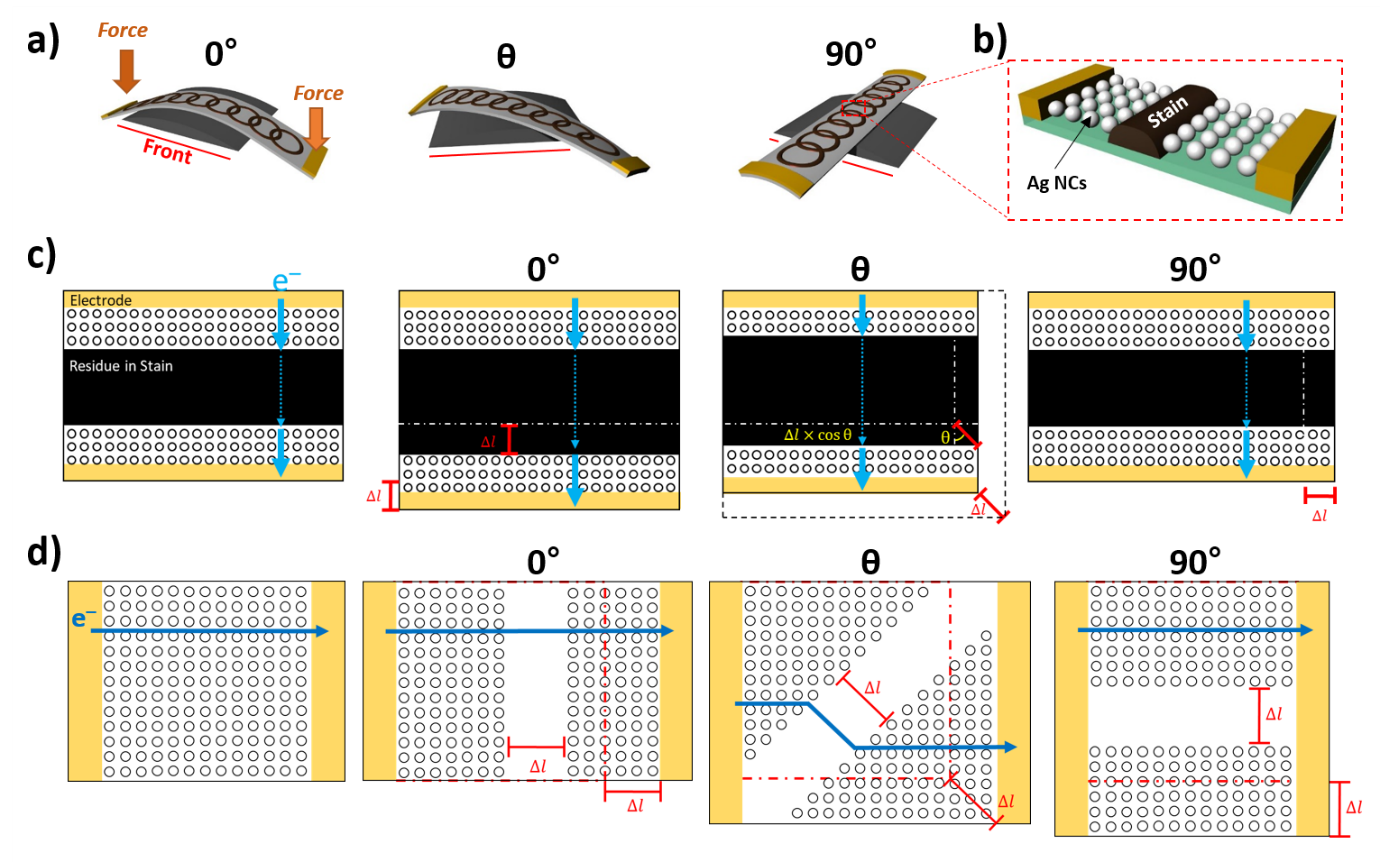
Fig. S7** **a** Schematic of films with alternately printed Ag NC patterns subjected to bending at different rotations. **b** Detailed schematic of alternately printed Ag NC patterns. Detailed top-view schematics of changes in the **c** alternately and **d** continuously printed Ag NC patterns with bending.

**
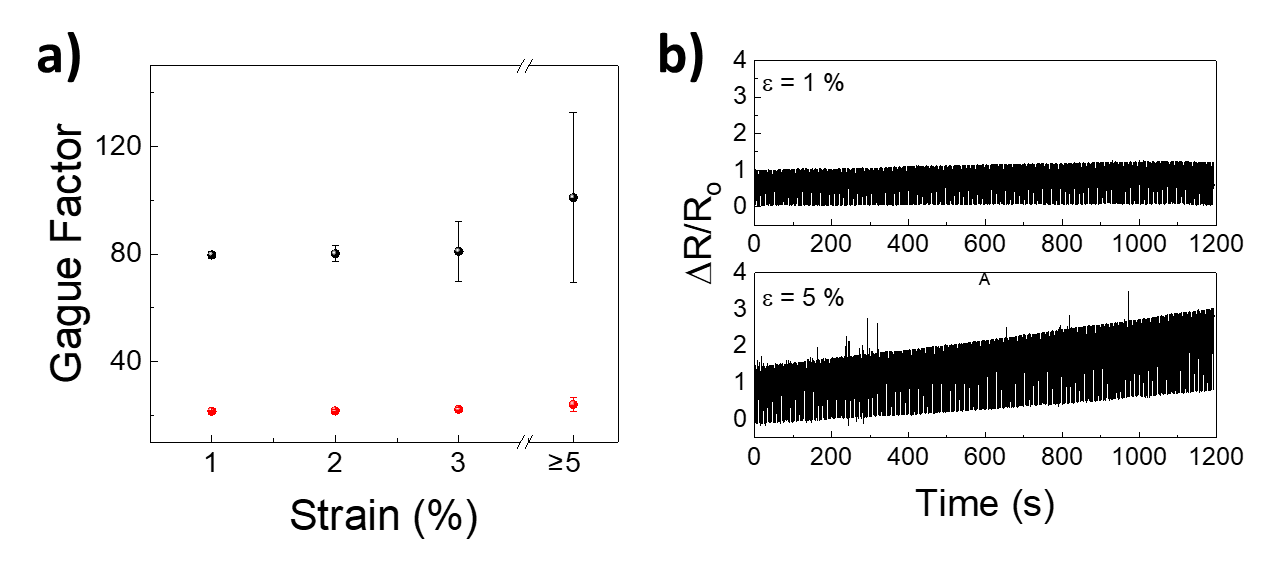
Fig. S8. a** Gauge factor of alternately- (black dots) and continuously printed Ag NC patterns upon high bending strain. **b** Cycle test of alternately printed Ag NC patterns (upper = 1% strain; lower = 5% strain)


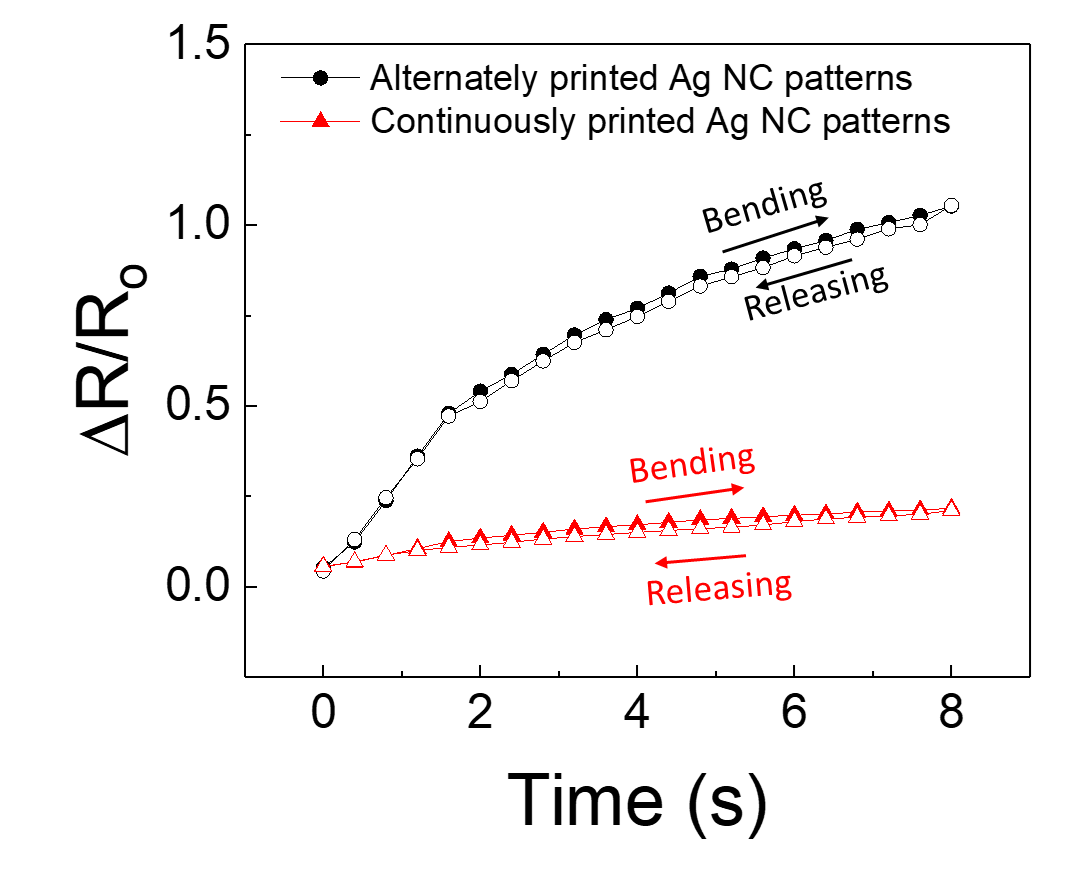
**Fig. S9**. Hysteresis plot of both Ag NC patterns with 1.0 % strain applied (filled circles or triangles) and released (vacant circles or triangles).

**References**

1. W. S. Lee, D. Kim, B. Park, H. Joh, H. K. Woo, Y. K. Hong, T. il Kim, D. H. Ha, S. J. Oh, Adv. Funct. Mater. **29**, 1806714 (2019)

2. D. Soltman, V. Subramanian, Langmuir **24**, 2224 (2008)
